# Botulinum Toxin Treatment of Psoriasis—A Comprehensive Review

**DOI:** 10.3390/toxins16100449

**Published:** 2024-10-18

**Authors:** Ali Ghaseminejad-Bandpey, Shahroo Etemadmoghadam, Bahman Jabbari

**Affiliations:** 1 Biggs Institute, University of Texas Health Science Center San Antonio, San Antonio, TX 78229, USA; ghaseminejad@uthscsa.edu (A.G.-B.); shahrooetemad@yahoo.com (S.E.); 2 Department of Neurology, Yale University School of Medicine, New Haven, CT 06516, USA

**Keywords:** psoriasis, plaque psoriasis, inverse psoriasis, nail psoriasis, botulinum toxin, botulinum neurotoxin, onabotulinumtoxinA, abobotulinumtoxinA, rimabotulinumtoxinA

## Abstract

A literature search on the subject of botulinum toxin treatment in psoriasis found 15 relevant articles, 11 on human subjects and 4 on animal studies. Of the human data, eight were clinical trials and three were single case reports. Seven out of eight clinical trials, all open-label, reported improvement in psoriasis following intradermal or subcutaneous botulinum toxin injections. One double-blind, placebo-controlled study, which used a smaller dose than the open-label studies, did not note a healing effect. Animal studies have shown that injection of botulinum toxins in the skin heals psoriatic skin lesions and can reduce the level of interleukins (ILs) and cytokines as well as inflammatory cells in psoriatic plaques. There is a need for controlled, blinded studies conducted in larger numbers of patients with doses that have shown promise in open-label studies.

## 1. Introduction

Psoriasis is a chronic autoimmune, inflammatory disease that primarily affects the skin and is often associated with significant comorbidities. It affects approximately 3% of the general population [1] with an equal distribution among men and women [2]. Psoriasis presents with a wide range of dermatologic features and involves a variety of body parts including the extremities, scalp, lower back, and sacral and palmoplantar regions as well as the nails and intertriginous areas such as the axilla, skin between digits, groin and breast. The four major clinical subtypes of psoriasis include plaque (most common), erythrodermic, postulate and guttate [3]. Mild psoriasis is currently treated with topical agents (corticosteroids, retinoids, calcineurin inhibitors, vitamin D preparations, PDE-4 inhibitors as well as phototherapy, whereas moderate to severe cases are preferably treated with biological agents introduced via subcutaneous injections or intravenous infusion [4,5].

**Figure 1:**
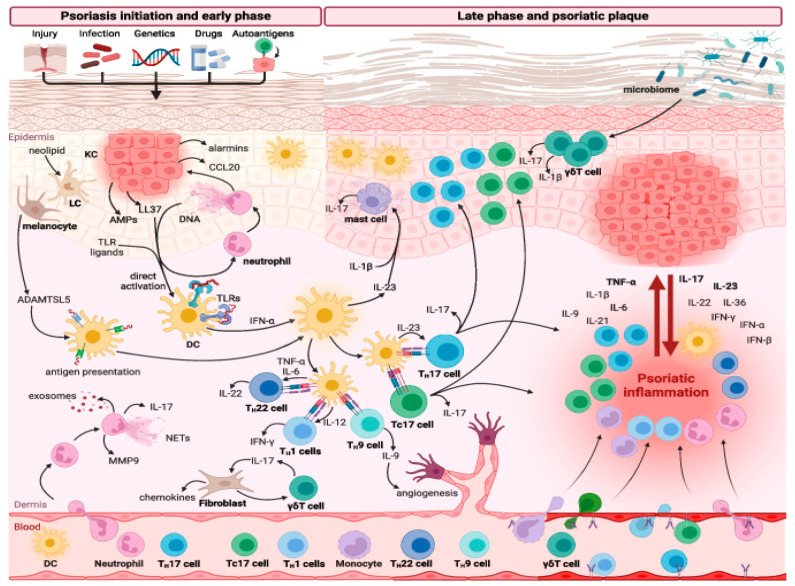
Dysregulation of the immune response in psoriasis. From Sieminska et al.—Clin Rev Allergy Immunol 2024 [8]. Reproduced under creative commons attribution—Courtesy of Springer publisher.

The etiology of psoriasis is complex and not fully understood. Genetic predisposition and epigenetic and environmental factors play a significant role; most importantly, dysregulation of the immune system has been implicated in its development, contributing to the diverse clinical manifestations and severity of the disease [6]. Research has shown that psoriasis is influenced by the intricate interactions between extracellular cytokine pathways and intracellular signaling molecules. Numerous cytokines convey signals from outside the cell to the cell membrane, where they bind to specific receptors. This binding activates intracellular signaling pathways, triggering a cascade of events that culminate in an inflammatory response [3]. Various inflammatory cells, cytokines and interleukins, including myeloid dendritic cells, Th1, Th17 and Th22 T cells, as well as IFN-α, IFN-γ, TNF-α, IL-1α/β, IL-23, IL-17 and IL-22, play crucial roles in the pathogenesis of this disease [7,8] (Figure 1).

Advances in understanding the mechanisms of psoriasis have led to the introduction of several new management options. Despite the revolutionary impact of biologic agents in treatment of psoriasis, they come with notable limitations, such as primary and secondary treatment failures, suboptimal initial responses, and exceptionally high costs. Similarly, topical agents have their own limitations, highlighting the need for more targeted treatment strategies to effectively manage the disease with minimal adverse effects [7].

Over the past 40 years, botulinum neurotoxin (BoNT) injections have been widely used in clinical medicine for different indications [9,10] and, more recently, in several areas of dermatology. They can be delivered effectively through intradermal and subcutaneous routes, are cost-effective with minimal side effects and offer diverse benefits, such as muscle relaxation, pain relief and anti-inflammatory effects [11,12]. Given that the primary goal in managing psoriasis is to reduce inflammation and clear skin lesions, BoNT injections hold promise as a potential therapeutic option for improving disease control and patient quality of life due to the latter effect.

The anti-inflammatory properties of BoNT have been demonstrated in both skin and extracutaneous conditions. BoNT/A has been shown to protect skin flaps, accelerate wound healing and reduce hypertrophic scars. In mouse models of psoriasiform dermatitis, BoNT/A improved symptoms, and it has also been effective in reducing inflammation in models of induced cystitis and arthritis by decreasing COX-2 and prostaglandin E2 receptor (EP4) expression, reducing monocyte and macrophage infiltration, and lowering IL-1β immunoreactivity [12]. Additionally, BoNT/A reduced pro-inflammatory M1 macrophages and downregulated LPS-induced activation of the JAK2/STAT1 and IκB/NFκB pathways, further supporting its potential as an anti-inflammatory agent [13].

Over the past 20 years, several publications have presented data in support of BoNT therapy in psoriasis. This comprehensive review is intended to furnish the data from the literature on the subject of BoNT therapy in psoriasis and explore the potential role and value of this form of treatment in the management of this disturbing medical disorder.

## 2. Study Design

**Figure 2:**
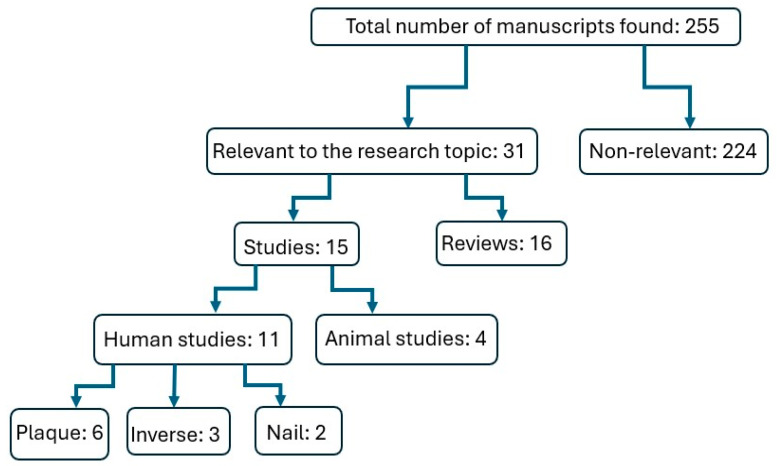
Flow chart of human and animal BoNT studies of psoriasis.

We reviewed the existing literature up to 1 July 2024 on BoNT therapy for psoriasis using the following search engines: PubMed, Scopus and Cochrane library. We used the following search words and phrases for this search: psoriasis, botulinum toxin, botulinum neurotoxin and psoriasis, onabotulinumtoxinA, incobotulinumtoxinA, abobotulinumtoxinA and rimabotulinumtoxinA. The search was conducted independently by two researcher/authors (BJ and AB). A third researcher/author (SEM) verified the results. The search results included only articles in the English language. Review manuscripts were excluded. The total number of articles found, the number of excluded items and the number of articles included in the final analysis are represented in a flow chart (Figure 2).

## 3. Results

This search disclosed 255 articles; of these, 15 were selected for final analysis (Figure 1). Eleven studies included data in humans (Table 1) and four were on animal models (Table 2). Of the 11 retrieved human studies, 1 was double blind and placebo-controlled, 7 were prospective and open-label while 3 were case reports.

Zanchi et al. [14] first reported that the injection of onabotulinumtoxinA can improve the appearance of skin lesions and complaints of patients with psoriasis. The authors screened 15 patients with inverse psoriasis before and at 2, 4 and 12 weeks after skin injections with onaA. Assessments included photography of the skin lesions and recording patients’ discomfort on a 0–10 pain scale (visual analogue scale—VAS). Authors used 50–100 units of the toxin applying multiple injections of 2.4 units per site, 2.8 cm apart. After 12 weeks, erythema extension, intensity and skin infiltration were all reduced significantly in 13 of 15 patients (documented photographically). The patients’ discomfort level in VAS fell from 9.1 to 2.1 after 4 weeks.

Three years later, in 2011, Saber and co-workers [15] using the same toxin and the same dosage (50–100 units), demonstrated marked clearing of axillary skin lesion in a patient with inverse psoriasis.

Gilbert and Ward [16] reported similar results in a case of plaque psoriasis injected intradermally with abobotulinumtoxinA (total 30 units, divided in 8 sites). The patient’s plaque severity improved in three weeks and the improvement was sustained for 7 months after injection. At 8th month post-injection, plaques gradually recurred at the same location. Tolberg and co-workers conducted a double-blind, placebo-controlled study in eight patients with plaque psoriasis [17]. Patients were injected with saline or abobotulnumtoxinA (a total of 36 units divided into 9 points) at the area of skin lesions. The primary objective of the study was a change in the sum of total clinical score (sum of erythema 0–3, scaling 0–3, infiltration 0–3, range 0–9) at 8 weeks. The authors noted no significant change at 8 weeks between toxin and saline injections.

In another two prospective, open-label studies, each including eight patients with plaque psoriasis, one using onA [18] and the other aboA toxin [19], investigators found toxin injections to be effective, leading to statistically significant improvements in the Psoriasis Area and Severity Index (PASI), Physician’s Global Assessment (PGA) and Total Critical Score (TCS) scores (Table 1). Gharib et al. [20] also reported a positive response after treating four patients with 50–100 units of aboA for inverse psoriasis as evidenced by significant reduction of both Eczema Area and Severity Index (EASI) as well as PASI scores after treatment (Table 1). Botsali and Erbil [22] reported significant improvement of nail discomfort and pain (a drop in VAS score of 4 points) in two patients with nail psoriasis after injection of 15 units of aboA (divided into two points) into the affected nail fold and the nail bed.

Two studies compared the effect of neurotoxin injection with another mode of treatment in psoriasis [21,24] (Table 1). In one [21], the effect of Renifex (a Chinese botulinum toxinA) was compared with the effect of 5-fluorouracil in 35 patients with plaque psoriasis. Renifex was injected once into psoriatic skin at multiple points 2.4 units/point, 2.8 cm apart. The total dose varied from 50 to 100 units depending on the extent of the lesion. Patients’ responses were assessed at 4 weeks post-injection. Assessments included Psoriasis Severity Scale (0–12 scale) and Psoriasis Disability Scale (a 15-item scale). Both treatment approaches significantly improved psoriasis. There was no statistically significant difference between the two treatments regarding efficacy and/or side effects.

The second study [24] compared the effect of abo-A injection for nail psoriasis with triamcinolone, vitamin D and no treatment over 24 weeks. The study was randomized, controlled and evaluator blind. There were 16 participants with 72 involved nail beds. The mean PASI of the studied population was 4.7. AboA (7.5 units) was injected once into each nail bed. Four nail beds were injected per patient. Triamcinolone injections were performed twice: once at the beginning of the study and once 8 weeks later. The applied dose was 0.05 mL/site (10 mg/mL). Patients’ nail appearance and Neuropathic Pain Severity Inventory (NPSI) were evaluated over time up to 24 weeks post-injection. With both injections, NPSI gradually decreased, reaching 40% reduction by week 16 post-injection. At 24 weeks post-injection, this reduction reached 60% in the aboA-injected group, whereas it remained at 40% in the triamcinolone-injected group. The difference between the two was statistically significant (*p* = 0.038). Side effects were infrequent and confined to local changes such as local pain after injection and, in one case, a small local hematoma.

One study [23] demonstrated significant improvement of psoriatic skin lesions following intramuscular injection of a large dose of aboA for spasticity. The patient previously had failed steroid and UV-B treatments.

## 4. Animal Studies

Our search found four studies on botulinum toxin treatment of psoriasis in animals (Table 2).

Ward et al. [25] investigated the effect of intradermal injection of abobotulinumtoxin A on psoriasis of KC-Tie2 Mice. In this murine model, overexpression of the angiopoietin receptor Tie2 in endothelial cells and keratinocytes leads to the development of a psoriasiform phenotype. Authors injected the dorsal skin of 11 mice affected by psoriasis with aboA (9 units/kg) and saline in two anatomically separate regions. The psoriatic skin area injected by aboA demonstrated significant reduction of acanthocytes compared with saline injected area at 2 and 6 weeks (*p* = 0.03 and 0.01, respectively). Furthermore, toxin-injected skin showed improvement in psoriasiform skin inflammation and epidermal hyperplasia. There was also notable reduction of infiltrating CD4+ T cells and CD11c+ DCs which coincided with reduction in acanthosis.

Amalia et al. [26] studied 10 mice that had developed psoriasis-like skin lesions after exposure to imiquimod (IMQ). The psoriatic skin was injected intradermally at four sites (2 units/site) with rimabotulinumB and compared with affected skin injected with normal saline. The rimaB-injected psoriatic skin showed significant reduction of epidermal thickness and erythema as well as reduction of the number of infiltrating CD3+, CD4+ T and CD11c+ DCs cells as well as infiltrating mast cells compared to the saline-injected psoriatic skin. The macrophages were also reduced in the rimaB-injected area, but the difference with vehicle-injected skin was not statistically significant. Other findings in rimaB-injected mice included reduction of some upregulated pro-inflammatory cytokines such as mRNA Il-17-A and IL-17 F as well as significant reduction of calcitonin gene-related peptide (CGRP) and substance P (SP) cells.

Chen et al. [27] applied 62.5 mg of 5% imiquimod cream to the dorsal skin of 6-week-old female C57BL/6 wild-type mice for 6 days. The resultant local psoriasis was then injected with one unit of onaA (divided into four sites), while the control group was injected with 50 microliters of normal saline. Compared with the saline-injected group, the onaA-injected group demonstrated alleviation of psoriasiform dermatitis manifested by decreased scaling and reduction of skin thickening. The PASI score was significantly lower in the onaA group compared to the control group (*p* < 0.05). Additionally, the number of both CGRP-positive neurons and secretion of CGRP in the culture diminished after subcutaneous injection of onaA into the psoriatic skin.

Huang at al. [28] emphasized the contribution of acid-sensing ion channels (ASICs) in psoriatic inflammation: classified as degenerin/sodium channels, they act as extracellular pH sensors. Among these channels, ASIC3 is strongly expressed in the peripheral nervous system and has been implicated in inflammatory and post-operative pain. Local injection of onaA into the mouse’s psoriatic skin reduces skin thickness, keratinocyte proliferation and local accumulation of cytokines in ASIC + mice, but not in the knocked-out (ASIC-) mice. Furthermore, local injection of onaA blocked the release of CGRP from acid-stimulated dorsal root ganglia (DRG) neurons in wild-type mice.

## 5. Discussion

Our search found 11 publications on human subjects relevant to the issue of botulinum toxin therapy in psoriasis, of which 8 were clinical trials. Albeit small in numbers of patients, seven of these eight studies demonstrated improvement of psoriasis after intradermal or subcutaneous injection of a neurotoxin [14,17,18,19,20,21,22,24] (Table 1). This positive observation was supported by three case reports that provided photographs of the healed skin after treatment [15,16,23]. However, one of the eight clinical trials, a controlled and blinded study on eight patients, reported lack of efficacy of BoNT treatment in psoriasis [17] (Table 1). The reason for this discrepancy is not clear. This blinded study was performed with abobotulinumtoxinA, whereas most of the other reported trials used onaA (Table 1). Although the units of BoNTs are not truly interchangeable, a ratio of 1 to 2.5 to 3 (1onaA unit approximating 2.5–3 aboA units) is generally accepted for clinical trials. Considering this ratio, 36 units of aboA used in the negative, blinded study translates to 12–14 units of onaA, a value that is far less than the 50–100 units used in the positive open-label trials (Table 1). Since in BoNT therapy the success of treatment is often dose dependent, this may be one explanation for the failure of this small double-blind trial.

The report of Popescu et al. [23] (Table 1) is interesting because they have shown improvement of skin psoriasis (photographically documented) after injection of 1000 units of abobotulinumtoxinA into different muscles for spasticity. The toxin must have diffused from the muscle into the skin to have resulted in this effect.

Psoriasis is an autoimmune disease with characteristic skin lesions resulting from a cascade of complex chemical and biological interactions. It is currently believed that this cascade of events starts with the activation of plasmacytoid dendritic cells (DCs). These cells secrete interferon 1 and alpha tumor necrosis factor (TNF). Alpha tumor necrosis factor promotes secretion of ILs 12 and 23. IL23 promotes proliferation of T-helper (TH17) cells that in turn increase secretion of IL17. IL12 increases differentiation of T-helper (TH1) cells. Local accumulation of these cytokines enhances proliferation of keratinocytes which along with infiltration of immune cells, increase angiogenic and adhesion factor expression leading to formation of psoriatic plaques [29]. Also, it has been shown that secretion of ILs (specifically IL23) is controlled by nociceptive nerve innervation of the skin; blocking the function of these peripheral nerve endings can prevent accumulation of cytokines in psoriatic skin lesions [30].

**Figure 3:**
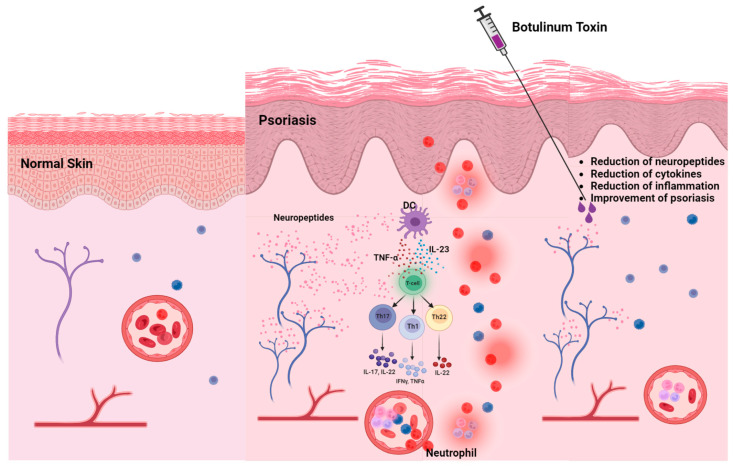
Injection of BoNTs into the skin inhibits the release of SP and CGRP, thereby attenuating dendritic cell activation and interrupting the downstream inflammatory cascade, ultimately leading to the reduction of keratinocyte hyperproliferation and clinical improvement of psoriatic lesions. Created in BioRender.com with permission.

Injection of botulinum toxins into the skin may heal psoriatic lesions through several mechanisms (Figure 3): Botulinum toxin-A can ameliorate microglial activated neuroinflammation as shown in adjuvant induced arthritic pain [31]. In a rat formalin model of inflammatory pain, injection of onaA into the rat’s paw before formalin injection reduces both formalin induced paw pain and local accumulation of inflammatory cells [32]. In murine models of psoriasis, injection of botulinum toxins (both A and B) reduces accumulation of CD4 and CD11 cells and IL17 within the psoriatic lesion [25,26], as well as decreasing the level of cytokines (Table 2). It has been proposed from animal studies that botulinum toxins may reduce local psoriatic inflammation by inactivating acid-sensing ion (sodium) channels (ASICs) [33]. Botulinum toxins can decrease secretion of cytokines and proliferation of keratinocytes by chemical denervation of the skin. Nociceptive nerve fibers use substance P and CGRP as their main pain transmitters. There is convincing evidence in the literature that both type A and type B toxins inhibit release of these neurotransmitters at peripheral nerve endings, dorsal root ganglia (DRG) cells and at the spinal cord level [33,34,35,36,37,38,39,40]. In a mouse model of psoriasis, investigators found decreased numbers of GCRP-positive cells after injection of onaA into the psoriatic skin and decreased secretion of CGRP in cell culture [27] (Table 2).

The molecular mechanisms through which botulinum toxins work on the nervous system have been the subject of numerous investigations and are currently well-defined for the motor system [41,42]. The molecular structure of botulinum toxin A consists of a light chain (50 KD) and a heavy chain (100 KD), linked together by a disulfide bond. After injection of the toxin into the tissue, the toxin molecule reaches the neuromuscular junction via the blood or lymphatics. The heavy chain attaches the whole toxin complex to the cell membrane and helps the toxin molecule enter into the cytosol. Once inside the cytosol, the disulfide bond breaks and the light chain, the active moiety of the toxin, through its enzymatic activity (protease), deactivates the intra-cellular SNARE proteins, hence inhibiting the release of acetylcholine from pre-synaptic vesicles. Although not studied in detail, there is some evidence that the function of BoNTs against sensory neurons is also conducted through blocking the function of SNARE proteins [43,44].

In recent years, the introduction of new biologic oral agents has revolutionized the treatment of moderate to severe psoriasis [29,45]. With the exception of etanercept that is a fusion protein acting as a soluble TNF receptor, all others are monoclonal antibodies (MCAs). Some of these MCAs (infliximab, adalimumab, certilozimab), like etanercept, work against TNF while others like ustekinomab, guselkumab, risankisumab and secokinumab specifically reduce the expression of different interleukins (IL12, IL17, IL23). The efficacy (75–80%) of these biologic agents against moderate to severe psoriasis has now been established through controlled and blinded studies and there are now studies underway to discern their long-term efficacy. Albeit uncommon, serious complications such as development of bacterial and fungal infections, different malignancies and congestive heart failure have been reported with the use of MCAs. Furthermore, it is recommended that before using MCAs for treatment of psoriasis, all patients should receive a course of anti-tuberculosis treatment; also, when using these drugs, vaccination with active vaccines should be avoided [29].

The advantage of BoNT therapy over treatment with MCAs is that serious complications are extremely rare with intradermal or subcutaneous injections when using FDA-approved toxins (onaA, incoA, aboA and rimaB). Moreover, the above-mentioned requirements and precautions (treatment with antituberculosis drugs before treatment of psoriasis and avoiding active virus vaccination) do not apply to BoNT treatment. However, the existing positive data on BoNT therapy for psoriasis are limited to small, uncontrolled, open-label clinical trials. Hence, proof of the efficacy of BoNT therapy in psoriasis awaits the results from well-designed, controlled, double-blind studies. These studies need to use high enough doses that have shown efficacy in the open-label clinical trials.

Several reviews have been published on the subject of botulinum toxin therapy for psoriasis. A great majority of these reviews have the title of botulinum toxin therapy in dermatological disorders with a small section devoted to psoriasis. The advantage of our review over the previous ones is that this review provides the most up to date information (up to July of 2024) on this subject. For instance, the last review of this subject published in 2021 [46], although comprehensive and informative, does not include the last six human studies depicted in Table 1 and provides no data on animal studies (our Table 2). Furthermore, in our review, we have provided a plausible explanation of why the double-blind, placebo-controlled study of Tolberg et al. [17] failed to show the efficacy of botulinum toxin therapy for psoriasis while all open-label clinical trials did—a point that we feel is important for readers to know.

The shortcoming of our review is that our search engines were limited to Medline, Cochrane library and Scopus as well as the fact that our review did not include data from non-English sources.

In conclusion, the existing literature in human subjects and animals suggests that botulinum toxin therapy is a potentially effective mode of treatment with a safe side effect profile for psoriasis. However, most reported studies include small numbers of patients with limited follow ups. The future perspectives in BoNT therapy for psoriasis include the conduction of well-designed, blinded studies in larger numbers of patients with longer follow-ups. Such studies should use doses that have shown to be effective in open-label studies.

## Figures and Tables

**Table 1:** Summary of human studies using botulinum toxin for the treatment of psoriasis.

Author and Date	Study Type	#pt	Type of Psoriasis	Type of Toxin	Dose	Results
Zanchi et al., 2008 [14]	Pro, OL	15	Inverse psoriasis	onaA	Total dose: 50–100 units, divided into 20–40 sites	Extension of erythema, intensity and infiltration showed improvement in 13 out of 15 subjects (87%). All patients reported symptom relief.
Saber et al., 2011 [15]	Case report	1	Inverse psoriasis	onaA	Total dose: 100 units	The patient experienced significant improvement of axillary psoriasis within one week of receiving the injections.
Gilbert & Ward, 2014 [16]	Case report	1	Plaque psoriasis	aboA	Total dose of 30 units divided in 8 sites.	Improvement of plaque severity within 3 weeks—complete remission after 7 months. Recurrence in 8th month.
Todberg et al., 2017 [17]	DB, PC	8	Plaque psoriasis	aboA	Total dose: 36 units, divided in 9 sites	Failed to improve the appearance of plaques.
Aschenbeck et al., 2018 [18]	Pro, OL	8	Plaque psoriasis	onaA	Total dose: 28–95 units	Injections were correlated with significantly reduced PASI and PGA scores (*p* < 0.01) for both scales.
González et al., 2020 [19]	Pro, OL	8	Plaque psoriasis	aboA	Total dose of up to 50 units, 5 units cm^3^	Significant improvement in TCS score in all subjects (*p* < 0.05) 4 weeks post-treatment.
Gharib et al., 2020 [20]	Pro, OL	4	Inverse psoriasis	aboA	Total dose: 50–100 units	Statistically significant reductions in both the EASI score and PASI score after treatment
Khattab & Samir, 2021 [21]	Refinex versus 5-fluouracil	35	Plaque psoriasis	Refinex	Total dose: 50–100 units	The response rate was 85% for Refinex and 90% for 5-FU (no significant difference). Side effect rate was similar. The recurrence rate was 15% for both agents.
Botsali & Erbil, 2020 [22]	Pro, OL	2	Nail psoriasis	aboA	Total dose: 15 units, divided in two sites	In both patients, VAS assessment of nail lesions improved by more than 4 grades.
Popescu et al., 2022 [23]	Case report	1	Plaque psoriasis	abo-A, IM for spasticity	Total dose: 1000 units	Marked improvement after a single trial in a patient who had failed responding to steroids and UV-B.
Juntongjin et al., 2024 [24]	Botulinum toxin versus triamcinolone acetonide and vitamin D	16	Nail psoriasis	aboA	Total dose: 30 units divided in 4 sites	One intralesional dose of BoNT-A delivered outcomes comparable to multiple TA injection sessions showing sustained effectiveness, especially in lesions affecting the nail bed.

EASI: Eczema Area and Severity Index; PASI: Psoriasis Area and Severity Index; TA: triamcinolone acetonide; PGA: Physician’s Global Assessment; VAS: Visual Analogue Scale; TCS: Total Critical Score (sum of erythema, desquamation and infiltration); onaA: onabotulinumtoxinA (Botox); aboA: abobotulinumtoxinA (Dysport); SC: subcutaneous; IM: intramuscular; Pro: prospective; OL: open label; DB-PL: double-blind, placebo-controlled; Refinex: Chinese botulinum toxin A not approved by the FDA; pt: patients.

**Table 2:** Animal research on botulinum toxin for the treatment of psoriasis.

Author and Date	Animal Model	#	Toxin	Dose	Assessed	Results
Ward et al., 2012 [25]	KC-Tie2 Mouse	11	aboA	9 units/kg intradermal injection, compared with saline	Histologic analysis and immunostaining for CD11c, CD4, F4/80, CD8	aboA injections led to significant reductions in psoriasiform skin inflammation and epidermal hyperplasia, as well as decreases in infiltrating CD4+ T cells and CD11c+ DCs, occurring simultaneously with improvements in acanthosis.
Amalia et al., 2021 [26]	Mice, imiquimod (IMQ) induced Psoriasis-like model	10	rimaB	2 units injected intradermally in 4 sites	PASI, histological examination by immunostaining, real-time RT-PCR	Marked decrease in PSI score, reduction of CD4, T cells, CD11c+ dendritic cells and IL-17A/F production in the lesion. Significant decrease in PGP9.5 and nerve fibers and neuropeptides.
Chen et al., 2022 [27]	Pre-treated IMQ model mice and Spinal hemi-sectioned mice	NS	onaA	Total: 1 unit (0.25)/site, subcutaneously	Immunofluorescence, histochemistry, western blotting, immunoelectron microscopy, qRT-PCR, ELISA, RNA sequence reanalysis	Skin injected by onaA showed less scaling and reduced erythema and thickness. PASI was significantly lower in onaA-injected skin than controls. Skin injected with onaA showed reduction of CGRP-positive cells and reduced secretion of CGRP in cell culture.
Huang et al., 2024 [28]	ASIC3 mice	NS	BoNT-A	30 units/kg	Immunofluorescence, histologic analysis of skin inflammation, CGRP release assay, flow cytometry, skin pH assessment	Reduced keratinocyte proliferation and epidermal thickening, decreased the elevated level of cytokines.

OnaA: onabotulinumtoxinA, rimaB; rimabotulinumtoxinB; PASI: Psoriasis Area and Severity Index; CGRP: calcitonin gene-related peptide; NS: not specified; ASIC3: acid sensing ion channel 3; qRT-PCR: quantitative reverse transcription polymerase chain reaction; ELISA: enzyme-linked immunosorbent assay; # number of studied animals.

## Data Availability

The data presented in this study are available in this article.
